# Pharmaceutical Composition of Hydrochlorothiazide:β-Cyclo-dextrin: Preparation by Three Different Methods, Physico-Chemical Characterization and *In Vivo* Diuretic Activity Evaluation

**DOI:** 10.3390/molecules16064482

**Published:** 2011-05-27

**Authors:** Maria Arlete Silva Pires, Robson Augusto Souza dos Santos, Rubén Dario Sinisterra

**Affiliations:** 1 Departamento de Química, ICEx, Universidade Federal de Minas Gerais, Avenida Pres. Antônio Carlos 6627, 31270-901, Belo Horizonte, Brazil; 2 Departamento de Fisiologia e Biofísica, ICB, Universidade Federal de Minas Gerais, 31270-901, Belo Horizonte, Brazil

**Keywords:** hydrochlorothiazide, β-cyclodextrin, intrinsic dissolution, diuretic

## Abstract

Hydrochlorothiazide is a common diuretic antihypertensive drug of the thiazide family. Its poor aqueous solubility is one of the reasons for its limited bioavailability after oral administration. This work aimed at the development of a hydrochlorothiazide:β-cyclodextrin (HTZ:β-CD) pharmaceutical composition in order to improve water solubility and bioavailability of the drug. The HTZ:β-CD complexes were prepared by three different methods: spray-drying, freeze-drying and fluid bed. Complexes were characterized by thermal analysis, Fourier transform-infrared (FTIR) spectroscopy, powder X-ray diffractometry, NMR (2D-ROESY), scanning electron microscopy (SEM), particle analysis and intrinsic dissolution. The findings reveal that three binary systems prepared presented better solubility results in comparison with free HTZ. Increased diuretic effect was observed to HTZ:β-CD obtained by fluid bed in comparison to free drug in rats. Results taken together suggest that pharmacological effect of HTZ in complex was increased by solubility improvement promoted by cyclodextrin.

## 1. Introduction

Hypertension remains a major clinical challenge worldwide because of both direct consequences of high blood pressure such as cerebral hemorrhage, hypertensive heart failure and progressive renal failure. In developed countries, heart disease and stroke are the first and the third-ranked causes of morbidity and mortality, respectively [1]. Pharmacological treatment of hypertension consists in the use of drug therapies including association or not of diuretics, beta-blockers, calcium channel blockers, angiotensin converting enzyme (ACE) inhibitors and angiotensin II receptor (AT1) antagonist (ARA) [1,2,3,4].

**Figure 1:**
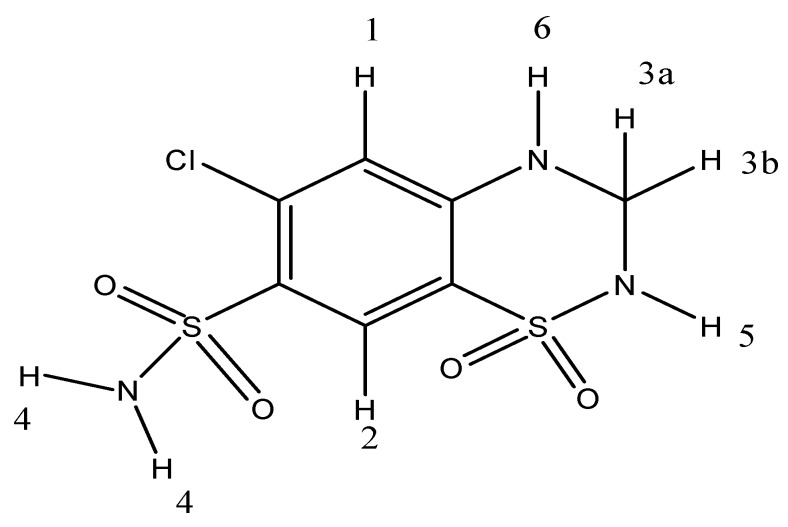
Structure of HTZ or 6-chloro-1,1-dioxo-3,4-dihydro-2H-benzo[e][1,2,4]-thia-diazine-7-sulfonamide(IUPAC nomenclature) [CAS number: 58-93-5].

Diuretics, in particular hydrochlorothiazide (HTZ) Figure 1, are often used in association with other drugs in the management of hypertension in patients with ischemic heart disease [5]. Thiazides affect the renal tubular mechanisms of electrolyte reabsorption, directly increasing excretion of sodium and chloride in approximately equivalent amounts. Indirectly, the diuretic action of hydrochlorothiazide reduces plasma volume, with consequent increase in urinary potassium loss, plasma renin activity, aldosterone secretion and decrease in serum potassium [6,7].

According to the Biopharmaceutics Classification System (BCS) aqueous solubility and permeability are the most important variables affecting drug bioavailability. HTZ is classified as Class IV, where the drugs have low solubility and low permeability characteristics after oral administration [8]. Cyclodextrins are one of the available pharmaceutical strategies in order to circumvent these drawbacks [9,10].

Cyclodextrins (CDs) are cyclic (α-1,4)-linked oligosaccharides of D-glucopyranose containing a relatively hydrophobic central cavity and a hydrophilic outer surface. CDs are able to form inclusion complexes with poorly water-soluble drugs. These inclusion complexes have been shown to improve stability, solubility, dissolution rate, and bioavailability [11,12,13]. This improvement in hydrophilicity may be attributed either to the formation of inclusion complexes or to the highly homogeneous assembly between CDs and drugs in the solid state. In most cases, this association increases the solubility of poorly soluble drugs. The drug-CD binary systems are also useful in dosage form development for increasing the solubility, dissolution, and absorption rates of poorly soluble drugs in tablet or capsule form [14].

One can find some works in the state-of-the-art that describe the use of cyclodextrins with HTZ in solution for self-assembly systems [15], and the use of CDs to increase the pharmacotechnical and dissolution parameters of pharmaceutical formulations [16].

To the best of our knowledge there are no descriptions of the preparation of HTZ:β-CD inclusion complexes by different methods and their complete physico-chemical characterization and diuretic evaluation in *in vivo* experimental models. Thus, the aim of this work was to investigate the effectiveness of β-CD containing systems in improving the solubility and the dissolution rate of HTZ. HTZ:β-CD complex binary systems were prepared by three different methods: spray-drying (SD), freeze-dried (FDY) and fluid bed (FB) in order to understand which of these methods could be used to prepare these inclusion compounds in a greater scale pharmaceutical production. Complexes were characterized by thermal analysis, Fourier transform-infrared (FTIR) spectroscopy, powder X-ray diffractometry, NMR (2D-ROESY), scanning electron microscopy (SEM), particle analysis, and intrinsic dissolution. *In vivo* experiments were performed in rats to evaluate the diuretic effects of the as prepared HTZ:β-CD complexes.

## 2. Results and Discussion

**Figure 2:**
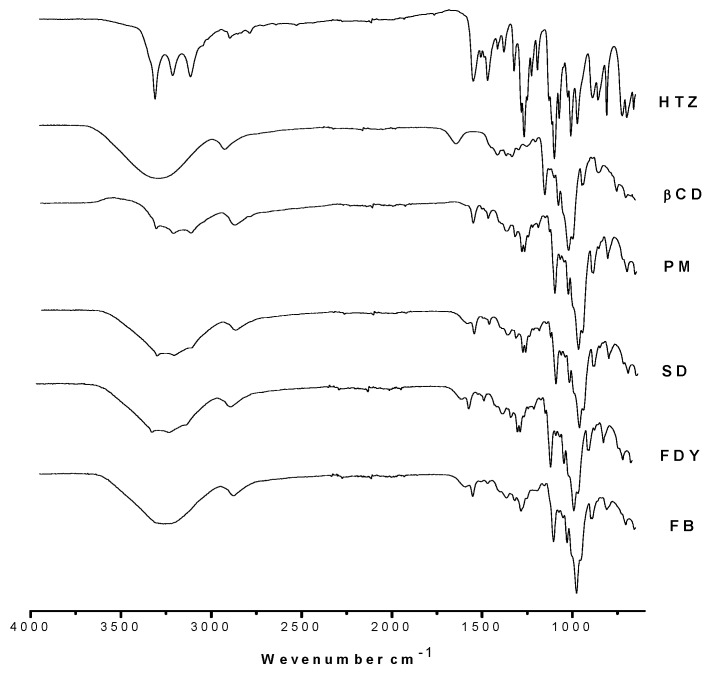
FTIR spectra for HTZ, β-CD, physical mixture (PM) and HTZ:β-CD complexes prepared by spray drying (SD), freeze-drying (FDY) and fluid bed (FB) methods.

Host:guest interaction in an inclusion complex is mediated by weak forces between molecules such as hydrogen bonds and hydrophobic interactions [3]. Figure 2 presents FTIR absorption spectra for HTZ, β-CD, physical mixture (PM) and HTZ:β-CD obtained by three different techniques. 

Comparison among HTZ, PM and the HTZ:β-CD complexes shows a loss of resolution in the typical HTZ bands observed at ν 3300 cm^−1^, $\bar{\nu }$ 1264 cm^−1^ and $\bar{\nu }$ 721 cm^−1^, corresponding to N-H_2_, SO_2_ and N-H bonds, respectively [17]. These results could be due to the host:guest interation between the HTZ aromatic moiety and the β-CD cavity. Disappearance of the O-H deformation band (δ _OH,_ ν 1640 cm^−1^) of water molecules in the β-CD cavity can be observed in the spectra of the PM and inclusion compounds. This fact may suggest complex formation by water loss of CD cavity and subsequent HTZ inclusion [18]. It can also be observed that the inclusion compounds’ spectra are very similar to each other, implying the three techniques used to obtain HTZ:β-CD are suitable to provide inclusion complexes.

**Figure 3:**
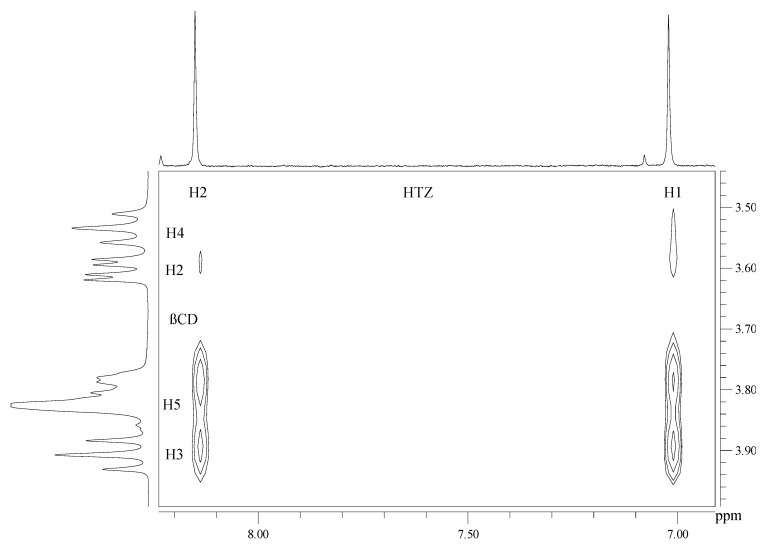
Partial contour map 2D-ROESY in D_2_O at 400MHz for HTZ:βCD prepared by the fluid bed (FB) technique.

In order to confirm the interactions between HTZ:β-CD observed by FTIR, NMR spectroscopy technique was also used. NMR spectroscopy of an inclusion compound between HTZ and β-CD has been previously described in the literature [19]. ^1^H-NMR spectra obtained for HTZ:β-CD demonstrated that there are correlations between HTZ and β-CD in complexes prepared by SD, FDY and FB. As shown in Figure 3, correlations occur among the H3, H5 and H6 of β-CD and the aromatic hydrogens H1 and H2 of HTZ in the complex prepared by FB. Further correlations can also be observed among H1 and H2 of HTZ and H2 and H4 of β-CD in the same complex, confirming the formation of the inclusion compound. Similar correlations to those observed for the complex obtained by FB were observed for the SD and FDY complexes.

**Figure 4:**
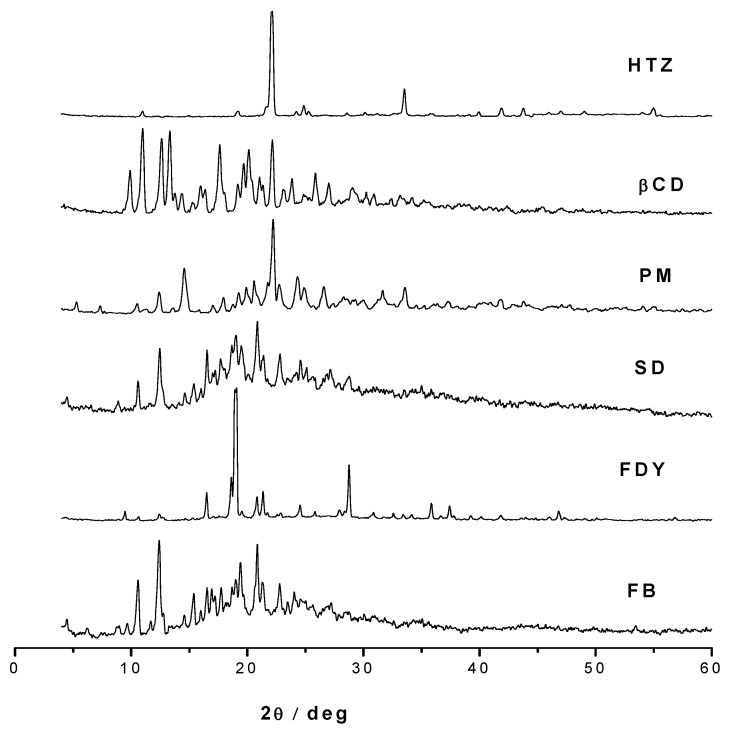
XRD diffractogram of HTZ, β-CD, physical mixture (PM) and the complexes prepared by spray drying (SD), freeze-drying (FDY), fluid bed (FB) methods.

Powder X-ray diffraction patterns of HTZ, β-CD, PM and three complexes are shown in Figure 4. It is well described in the state-of-the-art that the differences in the solid phases are responsible for the differences in drug’s solubility [20]. For example, an amorphous material is more reactive than a crystalline one due to its higher thermodynamic activity, and as consequence amorphous materials are considered more hygroscopic when compared to crystalline solids [20,21].

Thus, the XRD pattern diffraction studies are very useful in order to determine these properties; they do not provide, however, strong evidence of the formation of inclusion complexes. Analyzing the XRD pattern diffraction one can observe a semi-crystalline profile for the SD and FB inclusion complexes in contrast to crystalline XRD patterns diffraction the HTZ, β-CD, PM and FDY complexes. These results are in accordance to the higher solubility observed for these SD and FB complexes in comparison with the FDY complex since crystallinity and amorphicity are important factors that must be related to compounds’ solubility [20,21]. However, they are useful to monitor the compounds’ crystallinity changes upon host-guest interaction.

**Figure 5:**
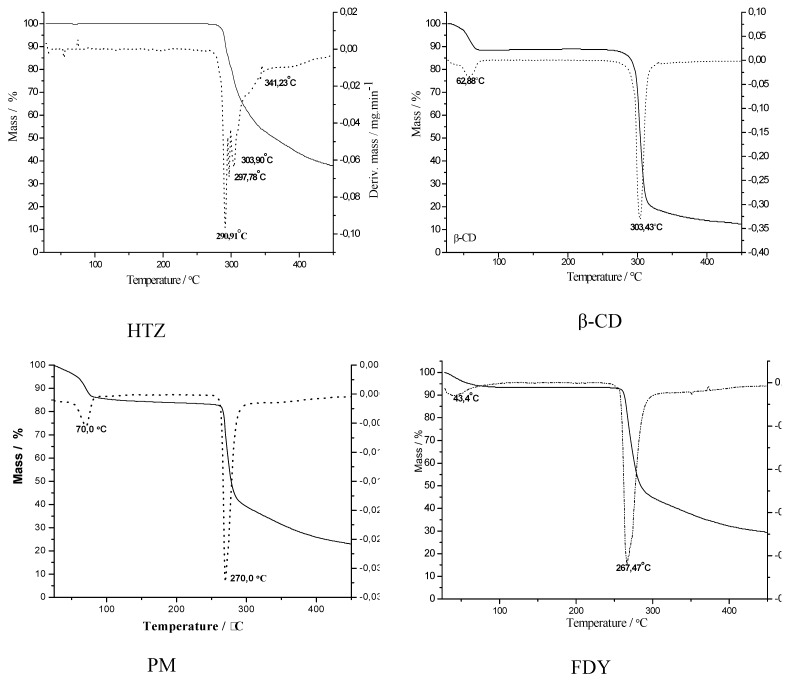

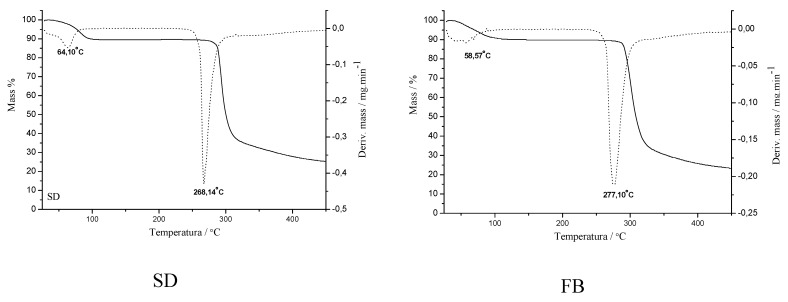
TG/DTG curves for HTZ, β-CD, physical mixture (PM) and the complexes prepared by spray drying (SD), freeze-drying (FDY) and fluid bed (FB).

Evidence of inclusion complex formation was obtained from thermal analysis. TG curves and their first derivative (DTG curves) of HTZ, β-CD, PM and the three inclusion complexes are shown in Figure 5. The HTZ curve presented a stable profile until about 290 °C, after which a weight loss of 50% was observed, suggesting an associated compound through hydrogen-hydrogen bonding interaction in the solid state. The β-CD TG curve showed two thermal events – one around 100 °C with 15% of mass loss, and the second in the range of 300-350 °C. These two thermal decomposition processes are in accordance with the literature [22] and could be associated to the loss of water molecules from the cyclodextrin cavity and the complete thermodecomposition of the β-CD, respectively. The PM curve showed a weight loss of 15% at about 70 °C, related with water loss from the cyclodextrin cavity, the same observed in the β-CD curve. 

Similar thermal decomposition profiles for the three HTZ:β-CD inclusion compounds were observed, where one can find two thermal events, one a 40-70 °C and the second from 260-270 °C; these events are associated to the loss of water and the thermal decomposition of the HTZ:β-CD complexes. It is interesting to note that these last TG profiles are very similar to those of the physical mixture, suggesting a limitation of the technique in this case, to monitor and distinguish an inclusion compound from a physical mixture.

**Figure 6:**
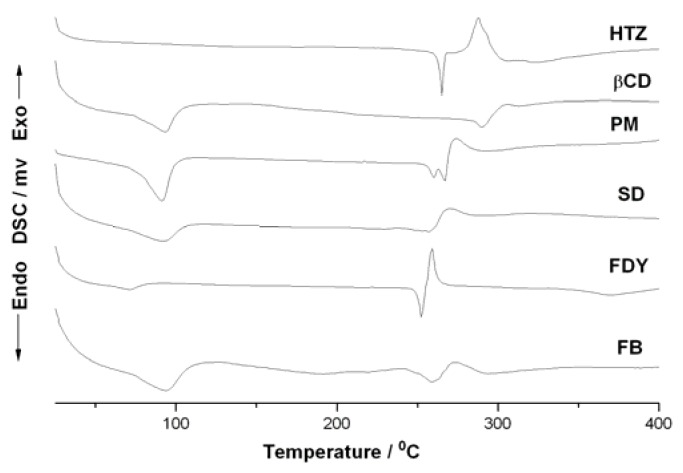
DSC curves for HTZ, β-CD, physical mixture (PM) and the complexes prepared by spray drying (SD), freeze-drying (FDY) and fluid bed (FB).

Figure 6 shows the DSC curves of β-CD, HTZ and for the three inclusion compounds. The DSC curve for HTZ presented two thermal events, one at about 270 °C and the second in 340 °C, that could be associated to the HTZ melting point and thermal decomposition, respectively [23]. Analyzing the β-CD DSC curve one can observe two endothermic peaks, one at 100 °C and the second around 300 °C. These events could be associated to the loss of water of β-cyclodextrin and the β-CD melting with decomposition, respectively. The PM DSC curve shows a typical profile for the thermal decomposition of a mixture where the individual phenomena of HTZ and β-CD are observed. Changes in the FB and SD’s DSC curves in comparison to HTZ, β-CD and PM were observed. The FB and SD’s DSC curves did not show the HTZ melting point at 270 °C, suggesting a host-guest interaction in both cases. Interestingly, the FDY’s DSC curve showed only a reduction of the HTZ melting point around 250 °C, suggesting the presence of a mixture between the inclusion compound and the physical mixtures in this case.

**Figure 7:**
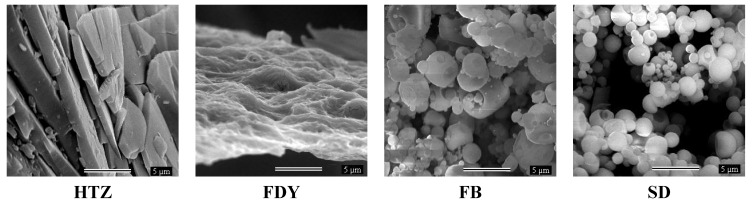
Photomicrographs of HTZ, and the complexes prepared by freeze-drying (FDY), fluid bed (FB) and spray drying (SD) methods, magnification 5000×.

Scanning Electron Microscopy (SEM) was performed for the raw materials and for SDY, FB and SD particles in order to investigate the morphology modification and the results are depicted in Figure 7. It could be observed that HTZ presented large and irregular crystals. Freeze-dried complex (SDY) showed a more amorphous solid with some porosity. In contrast, the FB and SD solid showed spherical particles with heterogenous size and also some surface porosity. However, the particles obtained by the FB technique did not present uniform size unlike what was observed for particles formed by SD. These results are in accordance with the literature where is described that the speed and drying time of the SD and FB techniques, could modify the morphology of the compounds obtained by these methods [21].

**Figure 8:**
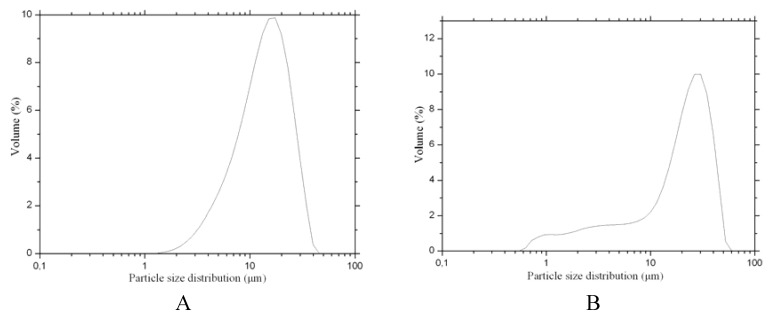

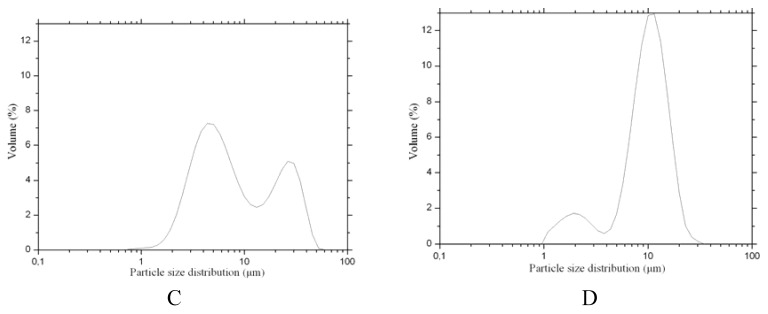
Particule size distribution, by number of particle, of the HTZ (**A**) and complexes prepared by freeze-drying (**B**), spray drying (**C**) and fluid bed (**D**).

Size distributions for HTZ and inclusion complexes are shown on Figure 8. Distribution curves suggested that HTZ (Figure 8A) has a unimodal particule distribution, presenting 90% of particle size below 27 μm. Higher HTZ particle size distribution it was observed in comparison with the respectively complexes particle size. SDY complex (Figure 8B) presented 90% of particle size below 32 μm. Besides, the SD and FB complexes (Figures 8C and 8D, respectively) have two particle size populations. In addition when the particle size distribution of the SD and FB complexes are analyzed one can observe that complexes show 90% of particles size below 17 μm and 32 μm, respectively (Figures 8C and 8D).

Dissolution studies are among of the most important *in vitro* tests during pre-formulation studies. They provide information about improvements in the bioavailability of drugs promoted by inclusion complexes. This trial do not only demonstrates the intrinsic solubility increments achieved by drug encapsulation but also allows the evaluation of the kinetics of drug release [24]. When applying the rotating disc method, the dissolution rate expression must be applied assuming laminar convective flow conditions and constant surface area, which is generally expressed as milligrams dissolved per minute per centimeter squared [25].

**Figure 9:**
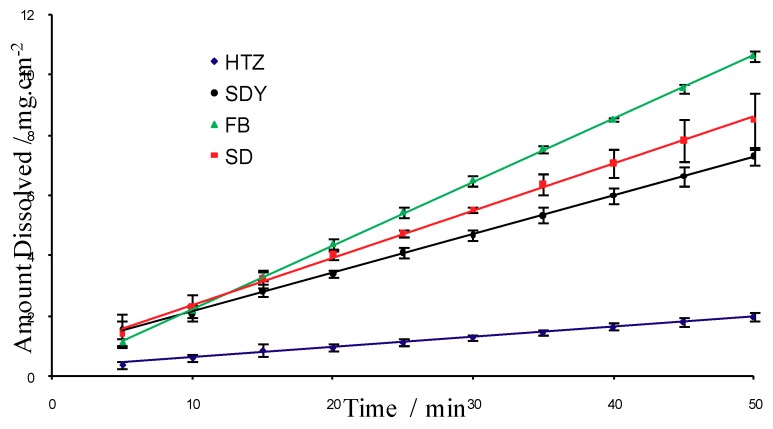
Intrinsic dissolution profiles for HTZ and complexes prepared by spray drying (SD), freeze-drying (FDY) and fluid bed (FB).

The dissolution profiles of HTZ alone and the three complexes in simulated gastric fluid (pH 1.2) are shown in Figure 9. Dissolution parameters, expressed as percent dissolved drug, and dissolution efficiency values at different times are presented in Table 1. The data were normalized to percentage of released HTZ versus time. Linearity was higher than 0.99 (Table 1) and the calculated intrinsic dissolution showed a Relative Standard Deviation (RSD) below 2% for HTZ and complexes, indicating acceptable reprodutibility. 

FB, SD and FDY exhibited dissolution rates up to 5.3, 4.3 and 2.4 times higher in comparison to the dissolution rate of HTZ. It is interesting to note that the observed higher dissolution rate for the FB and SD inclusion complexes could be due to the crystalline size and morphology of these compounds as discussed above, as one could obtain similar inclusion complexes with different crystalline profiles by different preparation methods. Powder granulometry and particle morphology of a compound are key factors for its dissolution rate [20]. Based on these results, one can suggest that the inclusion process has a key role in increasing the solubility of HTZ.

**Figure 10:**
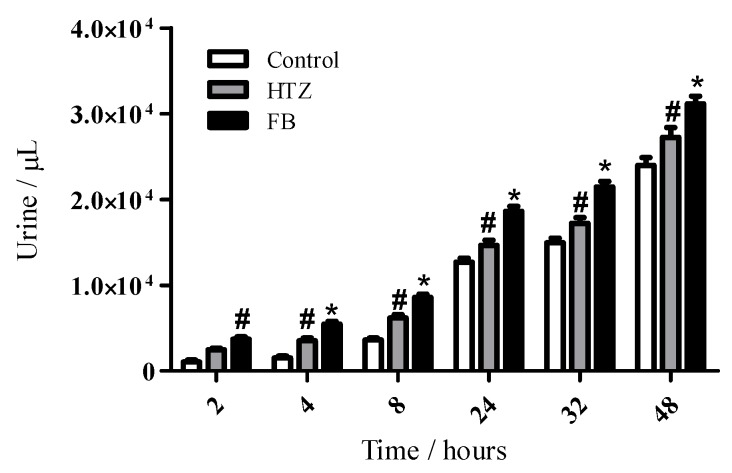
Time-course of urine output in Wistar rats treated with distilled water (control), HTZ and FB. The volume of excreted urine was measured at 2, 4, 8, 24, 32 and 48 h after the after oral administration of the compounds; cumulative values are reported as mean ± S.E.M for twelve rats in each group. # statistically different from control group and * statistically different from control and HTZ groups, p < 0.05.

Based on the physico-chemical characterization of the HTZ:β-CD inclusion compound by three different methods and their intrinsic dissolution profile we choose the FB HTZ:β-CD complex as a most interesting complexes in order to make the biological diuretic evaluation. It is interesting to mention that the quantification of ions such as sodium and chloride in urine is one of the best methods to determine the diuretic effect of drugs [6,7]. Results on the cumulative volumes of excreted urine after oral administration of the compounds are shown on Figure 10. 

**Figure 11:**
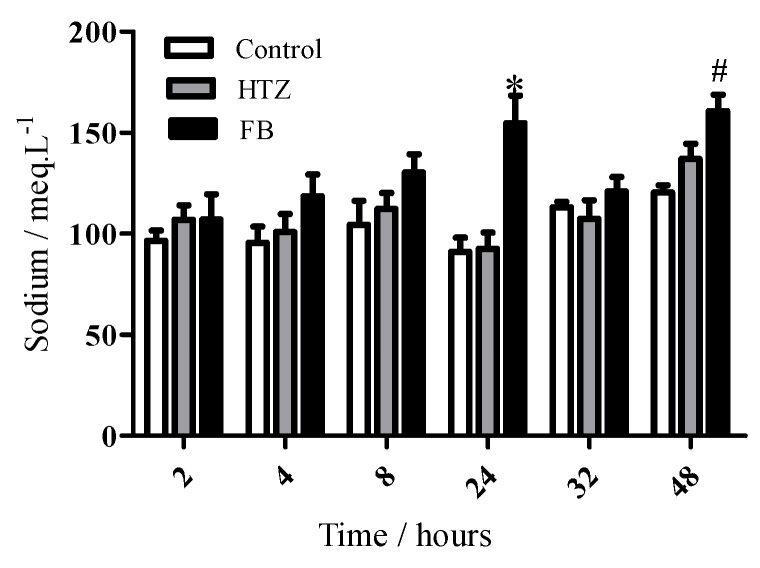
Time-course of excreted sodium in urine in Wistar rats treated with distilled water (control), HTZ and FB. The volume of excreted urine was measured at 2, 4, 8, 24, 32 and 48 h after oral administration of the compounds; cumulative values are reported as mean ± S.E.M for twelve rats in each group. # statistically different from control group and * statistically different from control and HTZ groups, p < 0.05.

A statistically different diuretic effect of HZT was observed after 4 hours in comparison to control and this effect was maintained until 48 hours. On the other hand, FB presented this effect from 2 hours after compound administration. In addition, the diuretic effect of FB was significantly different in comparison to HTZ and control between 4 and 48 hours. Figure 11 shows cumulative sodium values in excreted urine. Data showed a statistically different increase of the amounts of electrolyte in the FB group in comparison to the HTZ and control groups 24 hours after administration. This effect was also observed for FB in comparison to control after 48 hours. 

**Figure 12:**
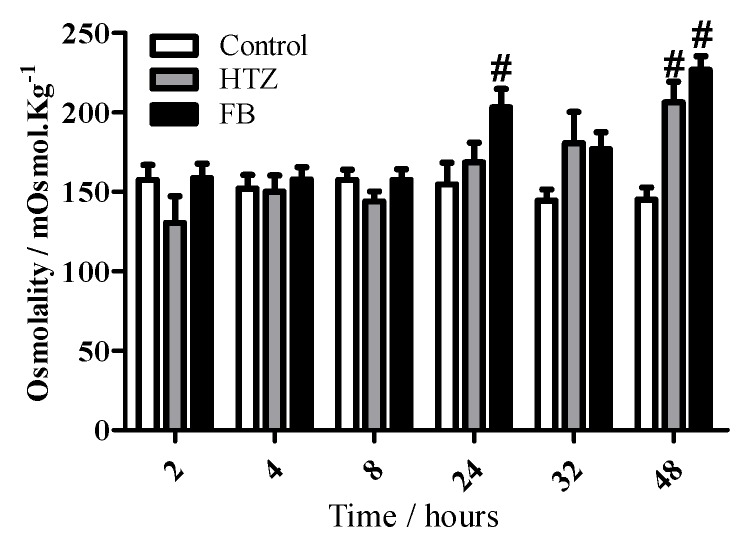
Time-course of urine osmolality in Wistar rats treated with distilled water (control), HTZ and FB. The volume of excreted urine was measured at 2, 4, 8, 24, 32 and 48 h after oral administration of the compounds; cumulative values are reported as mean ± S.E.M for twelve rats in each group. # statistically different from control group, p < 0.05.

Finally, osmolality values shown in Figure 12 are in accordance to sodium output values, since statistically significant differences were observed for HTZ (48 hours) and FB (24 and 48 hours) in comparison to control group.

Diuretic activities data of free HZT or associated to cyclodextrin suggested that CD increased the pharmacological effect of the drug in a time-course, considering equimolar dosis. This can be related to an increased solubility promoted by the FB formulation. Increased solubility profiles of cyclodextrin-containing formulations were previously reported in the literature for many drugs [3,9], including diuretics such as spironolactone [26]. Solubility is a crucial characteristic for increasing the bioavailability of drugs according to the BCS [27]. Therefore it was suggested that cyclodextrins and their inclusion compounds play an important role in the improvement of the bioavailability of low-solubility drugs such as HTZ, based on an increase of drug solubility and, probably, higher permeability. However the present work did not deal with the permeation experiments using cell models and this aspect will be developed in future studies. 

## 3. Experimental

### 3.1. General

HTZ (M_w_ = 297.74) was purchased from Ausun Chemical Co. Ltd and β-CD (M_w_ = ~1135.01) was purchased from Sigma-Aldrich (Milwaukee, WI, USA) and used as received. All other chemicals and solvents were of pharmaceutical or analytical reagent grade. The water was ultrafiltered by Milli-Q plus equipment from Millipore^®^ (Billerica, MA, USA).

### 3.2. Preparation of Inclusion Complexes

HTZ:β-CD inclusion complexes were prepared assuming a 1:1 stoichiometry and made using three different methods: spray-drying (SD), freeze-drying (FDY) and fluid bed (FB). The batches of SD, FDY and FB complexes gave 70, 90 and 60% yields, respectively.

#### 3.2.1. Freeze-Drying

HTZ (0.25g, 0.8 mmol·L^−1^) and β-CD (1.12 g, 0.98 mmol·L^−1^) were added separately in water (1 L) and submitted to heating (40 °C) with stirring until total dissolution. Subsequently, both solutions were stirred together for 4 hours. Resulting solution was frozen in liquid nitrogen and lyophilized (Savant Modulyo D-Freeze Dryer, Thermo Electron Corp., Waltham, MA, USA) for 72 hours. The obtained powder was stored at 4 °C.

#### 3.2.2. Spray-Drying

A Büchi model B290 laboratory-scale *spray-drier* was used. Solutions of HTZ and β-CD were obtained as described in the freeze-drying procedure. The mixture of both solutions was stirred for 4 hr and the obtained solution was subsequently atomized. The following conditions were used: airflow rate 30 m^3^/h, atomizing air pressure 1.0 Bar; inlet temperature 90 °C, corresponding to an out temperature 40 °C and flow rate of the solution 17 mL/min.

#### 3.2.3. Fluid Bed

A Mini Glatt *fluid bed* coater (Wurster insert, Glatt GmbH, Binzen, Germany) was used [21,28]. The solution containing a HTZ:β-CD (1:1) mixture was introduced into the fluid bed. The detailed operating conditions were as follows: inlet air temperature. 150 °C; product temperature 80 °C; air flow rate −30 m^3^/h; rate spray 30 mL/min; atomizing air pressure 1.5 bar; spray nozzle diameter 0.5 mm.

### 3.3. Fourier Transform-Infrared (FTIR) Spectroscopy

Infrared spectra covering the range of 4000-400 cm^−1^ were obtained with a Spectrum One FTIR spectrometer (Perkin Elmer, Waltham, MA, USA). The spectra were an average of 32 scans at resolution of 4 cm^−1^.

### 3.4. Nuclear Magnetic Resonance (NMR) 

NMR spectra were obtained and recorded on a DRX-400 Avance − 400 MHz spectrometer (Bruker-Biospin, Rheinstetten, Germany) at 300K. D_2_O was purchased from Aldrich and used as solvent, whose isotopic purity was at least 99.9% and tetramethylsilane (TMS) as internal standard (δ 0.0). The solutions were transferred to NMR tube with 8 inches in length and 5 mm in external diameter. One-dimensional NMR experiments (^1^H and ^13^C) were performed with 5 mm dual probe (^1^H/^13^C) using inverse detection with z-gradient coil. The intermolecular interaction between β-CD and HTZ was monitored by ^1^H-NMR and ^1^H-^1^H rotating-frame nuclear Overhauser spectroscopy, 2D-ROESY (500 ms spin lock). The water suppression was performed using the WATERGATE technique [29,30,31]. Data were processed using the software XWIN NMR, 3.1 (Bruker-Biospin, Rheinstetten, Germany) and edited with Mestre C^®^, version 4.9.9.6.

### 3.5. Powder X-Ray Diffractometry

X-Ray powder diffraction patterns were recorded at room temperature using a Rigaku Geigerflex 2037 from Rigaku Corp. (Tokyo, Japan). The measurement conditions were as follows: Co-filtered, Cu Kα radiation, scanning speed of 4θ per min over a 2θ range of 4° to 60°.

### 3.6. Thermal Analysis

Thermogravimetric analysis (TG) and Derivative Thermogravimetric analysis (DTG) analyses were performed on a Mettler TGA- SDTA 851 Star^e^ system (Mettler Toledo, Switzerland). Samples of about 4-6 mg were accurately weighted in open alumina pans and scanned from 25 °C to 450 °C, using a 2 °C·min^−1^ heating rate, under nitrogen atmosphere (50 mL·min^−1^). The instrument was calibrated with aluminum and indium as standards.

Differential Scanning Calorimetry (DSC) curves were produced in triplicate in a DSC Mettler 822 Star^e^ system (Mettler Toledo, Switzerland) using the following conditions: dynamic nitrogen atmosphere (50 mL·min^−1^), heating rate of 2 °C·min^−1^. Samples of about 2-3 mg were weighed out accurately and submitted to further heat scanning from 25 °C to 450 °C in a sealed aluminum pan with a capacity of 40 μL. An empty sealed aluminum pan was used as reference. The equipment was periodically calibrated with indium (99.98%, m.p. 156.65 °C, Sigma-Aldrich, Milwaukee, WI, USA). 

### 3.7. Scanning Electron Microscopy (SEM)

The surface morphology of pure components and their equimolar binary systems obtained by different methods were examined by means of a JEOL (JSM 840 A, 4-10 Kv model) scanning electron microscope. The powders were previously fixed on a brass stub using double-sided adhesive tape and then were made electrically conductive by coating, in a vacuum, with a thin layer of gold (100-300 Ǻ), for 240 s. Photographs were taken at an excitation voltage of 10 Kv and appropriate magnifications.

### 3.8. Particle Size Analysis

The ground mixture was dispersed in vegetable oil and the suspension was sonicated for 5 min. The particle size distributions were determined by the laser diffraction technique with a Malvern Mastersizer 2000 instrument (Malvern Instruments, United Kingdom).

### 3.9. Intrinsic Dissolution

The dissolution studies were conducted under sink conditions in HCl solution (0.1 mol·L^−1^, 900 mL) at 37 ± 0.5 °C and rotational speeds of 100 rpm. Each dissolution test was performed at least in quadruplicate [25]. The dissolution system was fitted with a ERWEKA DT800 (Distek Inc., NJ, USA) and a HP 89092A 7-channel peristaltic pump (Agilent Technologies Italia Spa., Roma, Italy). The collected aliquots containing HTZ were filtered using a 0.45 μm filter (Millipore^®^), and analyzed in a spectrophotometer at 270 nm.

Discs with FDY, SD, FB complexes and HTZ were prepared compressing powder (200.0 mg) in a Perkin Elmer hydraulic press (Waltham, Massachusetts, USA) for 1 min under 3.5 t compression force, using a 8 mm punch. The surface area exposed was 0.5 cm^2^ and the disk distance from vessel bottom was 2.54 cm. Aliquots of HTZ from the mixtures prepared by the FDY, SD, FB methods were automatically collected every 5 minutes until 50 minutes. The results were normalized to the percentage of HTZ released, a linear regression of HTZ released versus time was plotted and the intrinsic dissolution rate of the drug was determined in mg per minute per cm^2^ from the slope of the regression line calculated. Only the linear portion of each dissolution profile was considered for the intrinsic dissolution rate determination. The slope and the other statistical parameters of the curves were calculated by linear regression analysis.

### 3.10. Evaluation of the Activity Diuretic of HTZ and FB Complex

Male Wistar rats (300-350 g) were obtained from the animal facilities of ICB (CEBIO), UFMG. The rats were housed individually in metabolic cages, controlled conditions of temperature (25 °C) and a 12:12 h light/dark cycle. Studies were performed in accordance with the guidelines for the human use of laboratory animals of our institution and approved by local authorities.

Diuretic efficacies *in vivo* of HTZ and FB were evaluated in comparison to control. After 48 hr adaptation/acclimatization in a metabolic cage, the animals were randomized in three experimental groups: control (n = 4), a HTZ (n = 4) and a FB (n = 4) one. Suspensions of HTZ and FB were prepared in distilled water and administered to animals by gavage performing doses of 10 mg·kg^−1^ body weight (HTZ) and 38 mg·kg^−1^ body weight (FB, equivalent to 10 mg·Kg^−1^ of HTZ) [32,33]. Distilled water (H_2_O) was used as control.

**Figure 13:**
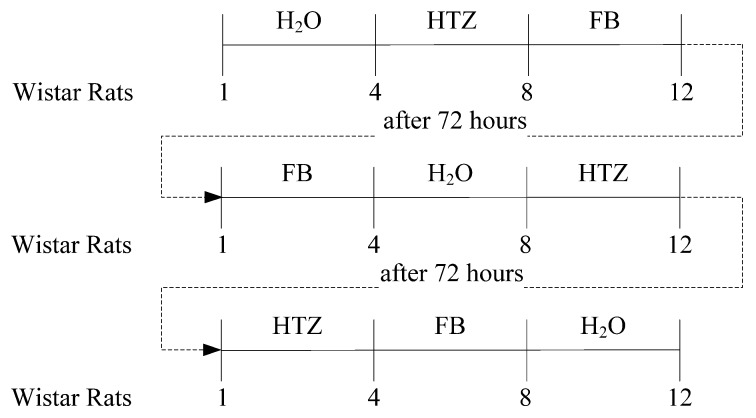
Study scheme. The wistar rats were randomized in three experimental groups with four rats each one. Oral administration by gavage: 1 mL of distilled water (control); HTZ (10 mg·Kg^−1^ body weight) and FB (38 mg·Kg^−1^ body weight). Inversion of groups occurred 72 hours after administration.

The three groups of rats were allocated to one of three different treatments as summarized in Figure 13. The period of cumulative urine output was recorded at 2, 4, 8, 24, 32, and 48 hr after oral administration of compounds. The urine volume was measured and a urine sample was taken for further analysis. Urinary sodium was determined in a flame photometer (CELM FC-180, Belo Horizonte/MG, Brazil). Osmolality were measured in a osmometer (MicroOsmette, Natick, MA, USA) Results were presented as mean ± S.E.M. (standard error of mean) and were analysed by two-way analysis of variance followed by Boferroni *post hoc* test when the main effect was significant. A p < 0.05 was considered significant.

## 4. Conclusions

Based on the results one can conclude that all the three methods proposed in this work are efficient for obtaining HTZ:β-CD inclusion compounds, but it is interesting to note that among them the most promissory method is the fluid bed one. This method could be easily used for the pharmaceutical industrial production of inclusion complexes using cyclodextrins. 

Higher intrinsic dissolution of the inclusion compounds in comparison to the free HTZ was also demonstrated. Better diuretic activity for the HTZ included in cyclodextrin was obtained by the fluid bed method in comparison with the free HTZ, a result that could be due to the higher water intrinsic dissolution and changes in the crystalline size and morphology introduced by the cyclodextrin on the host:guest were interesting.

Enhanced pharmacological effect of HTZ in the FB formulation can be related to an improvement on oral bioavailability, as a consequence of the increased solubility, which makes the CDs important pharmaceutical excipients. 

## Figures and Tables

**Table 1:** Linear regression, linearity and intrinsic dissolution for HTZ and complexes prepared by spray drying (SD), freeze-drying (FDY) and fluid bed (FB).

	Linear Equation	Linearity	Intrinsic Dissolution Efficiency (mg·min^−1^·cm^−2^)
**HTZ**	Y = 0.0345x + 0.2718	0.9960	0.040 ± 0.003
**SD**	Y = 0.1571x + 0.7908	0.9986	0.170 ± 0.018
**FDY**	Y = 0.1287x + 0.8545	0.9996	0.146 ± 0.005
**FB**	Y = 0.2109x + 0.1278	0.9998	0.213 ± 0.004
